# Supplementary material for: Thermoelectric coupling effect in BNT-BZT-xGaN pyroelectric ceramics for low-grade temperature-driven energy harvesting
Source: Nat Commun. 2023 Nov 30;14:7907. doi: 10.1038/s41467-023-43692-3 (PMC10689474; doi:10.1038/s41467-023-43692-3)
Supplement: Supplementary file 1 — Supplementary Information [file 41467_2023_43692_MOESM1_ESM.pdf]

Supporting Information

Thermoelectric coupling effect in BNT-BZT-*x*GaN pyroelectric ceramics for low-grade temperature driven energy harvesting

Meng Shen[1,2*], Kun Liu[1], Guanghui Zhang[1], Qifan Li[1], Guangzu Zhang[3], Qingfeng Zhang[1,4*], Haibo Zhang[5], Shenglin Jiang[3], Yong Chen[1*], Kui Yao[2*]

[1] Hubei Key Laboratory of Micro-Nanoelectronic Materials and Devices, Hubei Collaborative Innovation Center for Advanced Organic Chemical Materials, Ministry of Education Key Laboratory of Green Preparation and Application for Functional Materials, and School of Microelectronics, Hubei University, Wuhan 430062, China

[2]Institute of Materials Research and Engineering (IMRE), A*STAR (Agency for Science, Technology, and Research), Singapore, 138634 Singapore

[3]School of Optical and Electronic Information and Wuhan National Laboratory for Optoelectronics, Huazhong University of Science and Technology, Wuhan, Hubei 430074, China

[4] Ministry of Education Key Laboratory of Green Preparation and Application for Functional Materials, Hubei Key Laboratory of Micro-Nanoelectronic Materials and Devices, Hubei Key Laboratory of Polymer Materials, School of Materials Science & Engineering, Hubei University, Wuhan 430062, China

[5]School of Materials Science and Engineering, State Key Laboratory of Material Processing and Die & Mould Technology, Huazhong University of Science and Technology, Wuhan 430074, China

[*]E-mail: sm@hubu.edu.cn, zhangqingfeng@hubu.edu.cn, chenyong@hubu.edu.cn, k-yao@imre.a-star.edu.sg

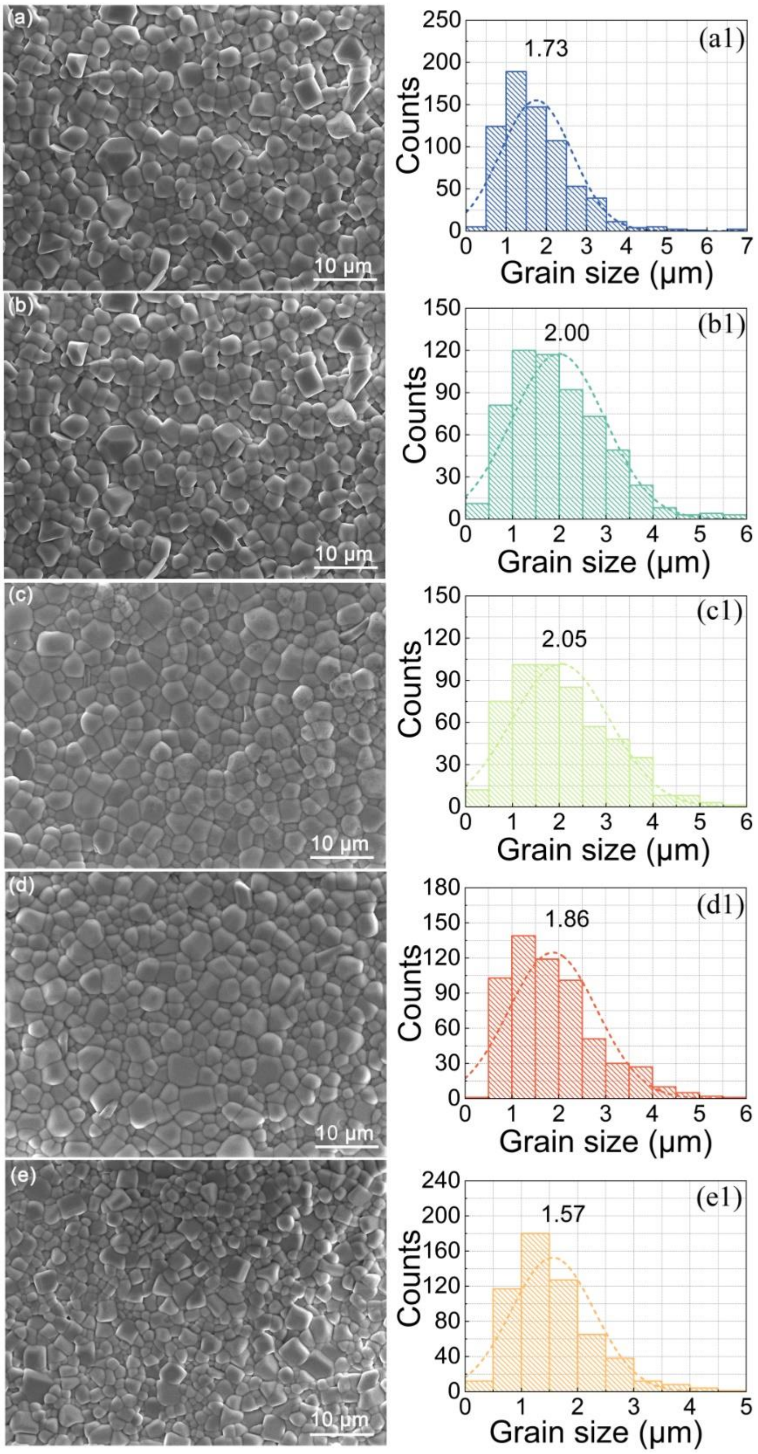

Supplementary Fig. 1 The microstructure measured by FE-SEM and the average grain size calculated by the Nano Measurer software. (a)-(e) The SEM morphology of BNT-BZT-*x*GaN with *x*=0-0.2 wt%, (a1)-(e1) the average grain size of BNT-BZT-*x*GaN with *x*=0-0.2 wt%.

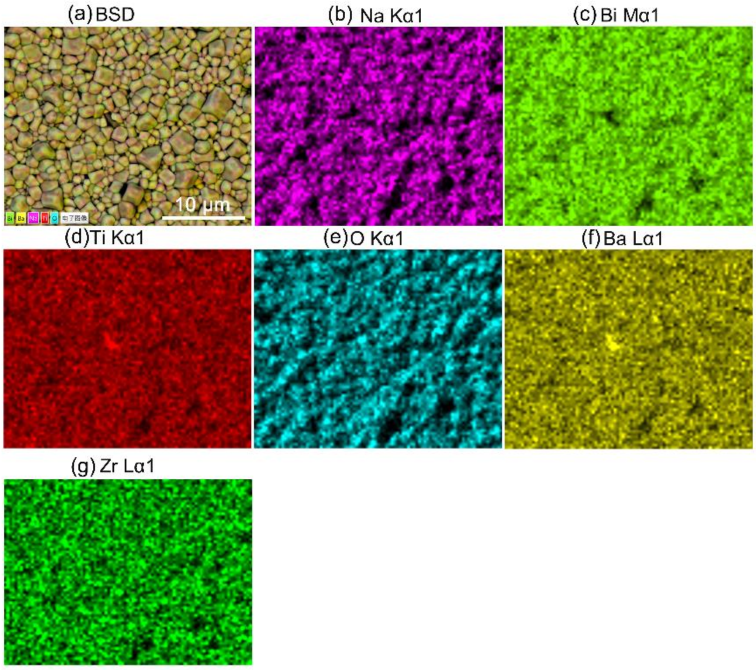

Supplementary Fig. 2 Compositional heterogeneity and microstructure measured by FE-SEM and EDS. (a)-(g) The backscattering diffraction (BSD) image and the corresponding elemental distribution of BNT-BZT ceramics.

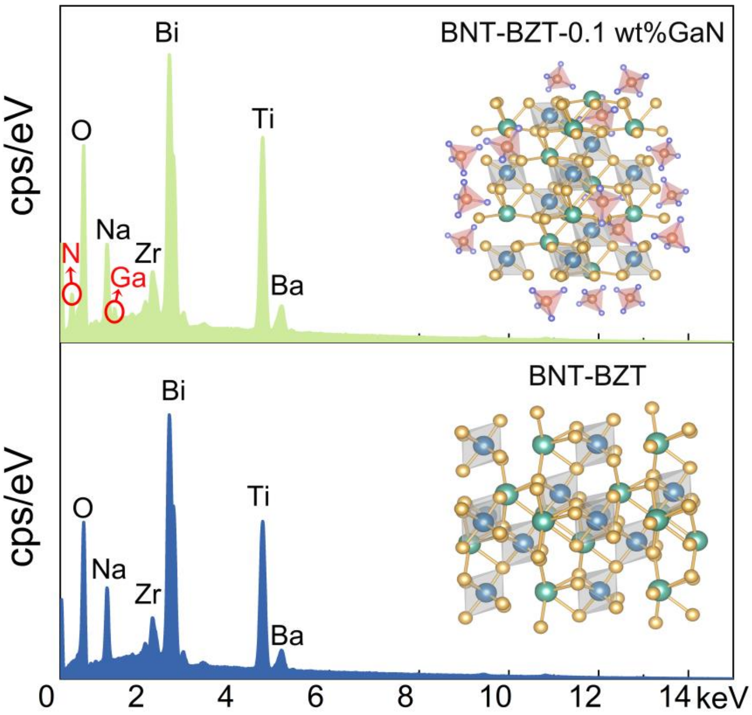

Supplementary Fig. 3 Compositional heterogeneity measured by EDS. The energy-dispersive spectrum patterns of BNT-BZT and BNT-BZT-*x*GaN with *x*=0.1 wt%

ceramics.

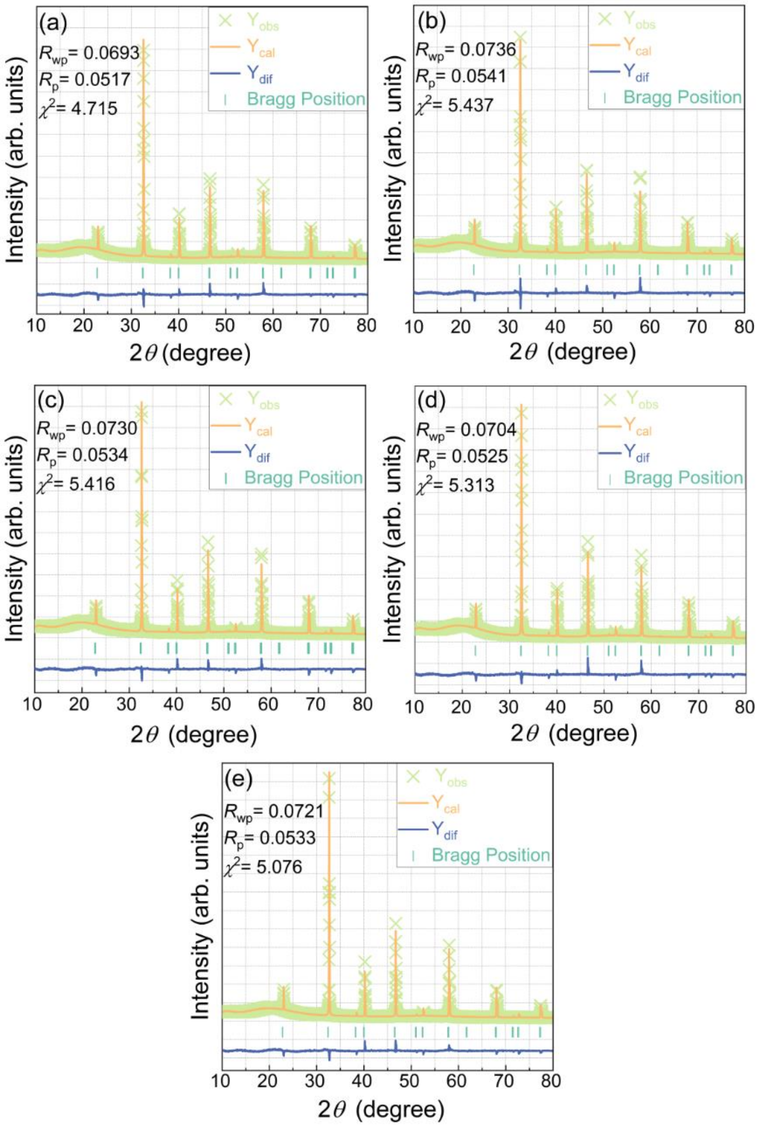

Supplementary Fig. 4 The XRD Rietveld refinements of BNT-BZT-*x*GaN with various contents of GaN calculated by the GSAS-EXPGUI software. The XRD patterns of BNT-BZT-*x*GaN ((a) *x*=0, (b) *x*=0.05 wt%, (c) *x*=0.1 wt%, (d) *x*=0.15 wt%, (e) *x*=0.2 wt%).

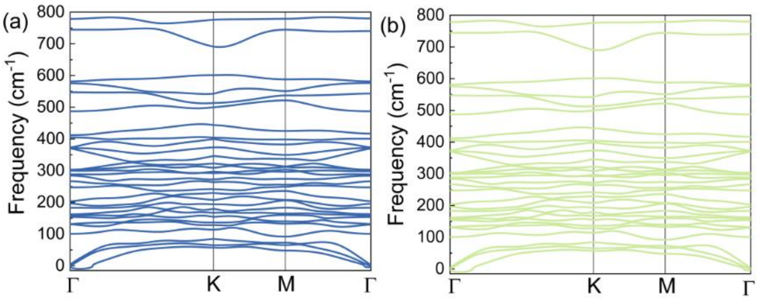

Supplementary Fig. 5 The theoretical calculation of the phonon structure. The phonon-dispersion curves along the high-symmetry lines of Brillouin zone of (a) BNT-BZT and (b) BNT-BZT-*x*GaN with *x*=0.1 wt%.

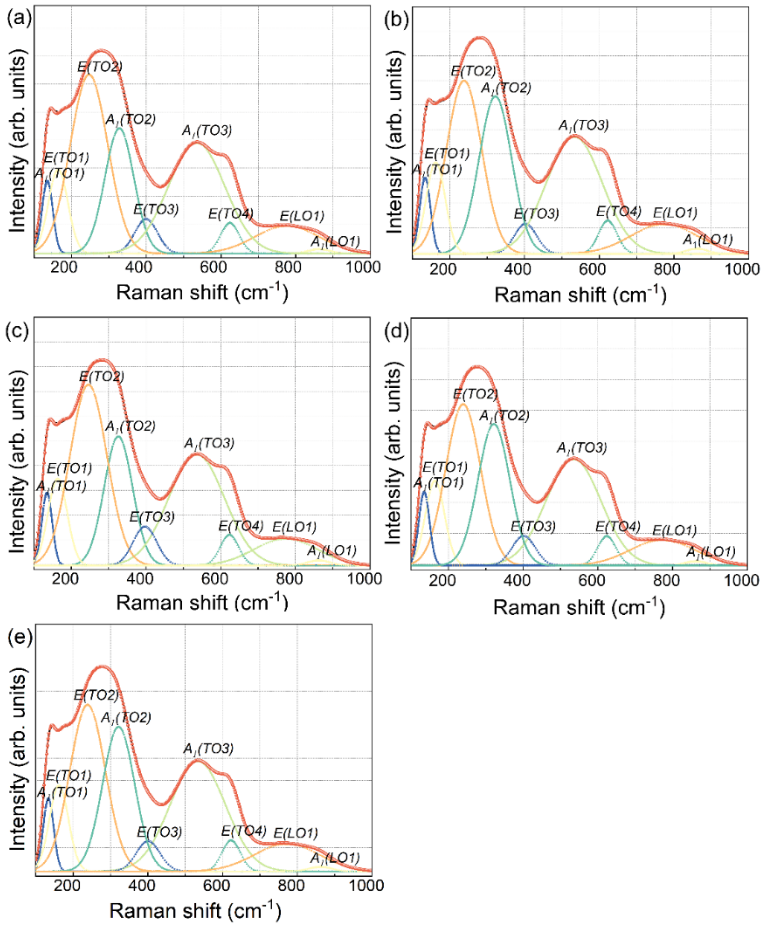

Supplementary Fig. 6 The Raman spectra of BNT-BZT-*x*GaN ceramics measured by laser Raman spectrometer. The Raman spectra of BNT-BZT-*x*GaN((a) *x*=0, (b) *x*=0.05 wt%, (c) *x*=0.1 wt%, (d) *x*=0.15 wt%, (e) *x*=0.2 wt%). These frequency modes are divided into three parts include low (below 200 cm^{-1}), middle (200~600 cm^{-1}) and high (600~900 cm^{-1}) wavenumbers regions. For the BNT-BZT-*x*GaN ceramics, the Raman mode at low frequency is derived from the vibrations of A-site positive ions (Na^{+}, Bi^{3+}). In the middle wavenumbers regions, the intense frequency modes originate to O-Ti motion and vibrations of the TiO_6 octahedra. Finally, the high-frequency vibrations between 600 cm^{-1} and 900 cm^{-1} are linked to vibrations involving oxygen displacement in TiO_6 octahedra.

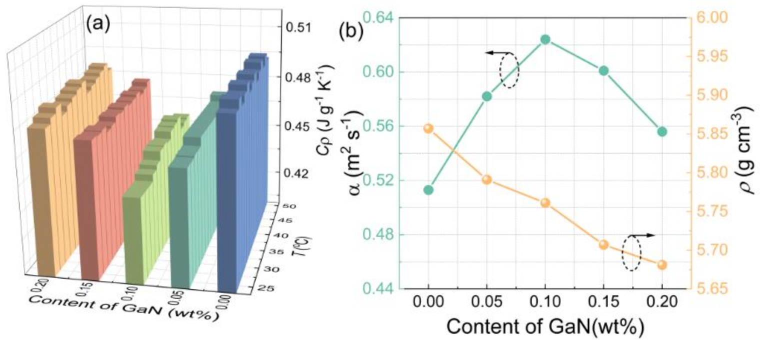

Supplementary Fig. 7 Thermal transport. (a) The temperature-dependent specific heat capacity measured by DSC, (b) The thermal diffusivity and the density of BNT-BZT-xGaN ceramics with different GaN measured by a LFA427 Microflash.

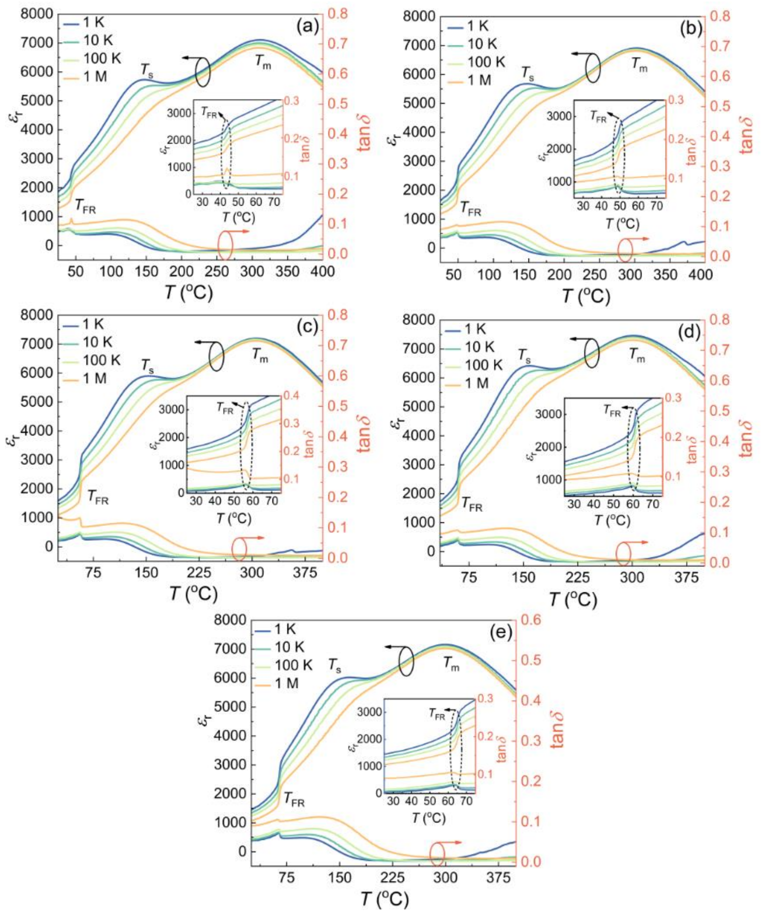

Supplementary Fig. 8 Dielectric properties measured with Dielectric Properties Testing System. The temperature dependence of ε_r and tanδ for BNT-BZT-xGaN ceramics ((a) x=0, (b) x=0.05 wt%, (c) x=0.1 wt%, (d) x=0.15 wt%, (e) x=0.2 wt%).

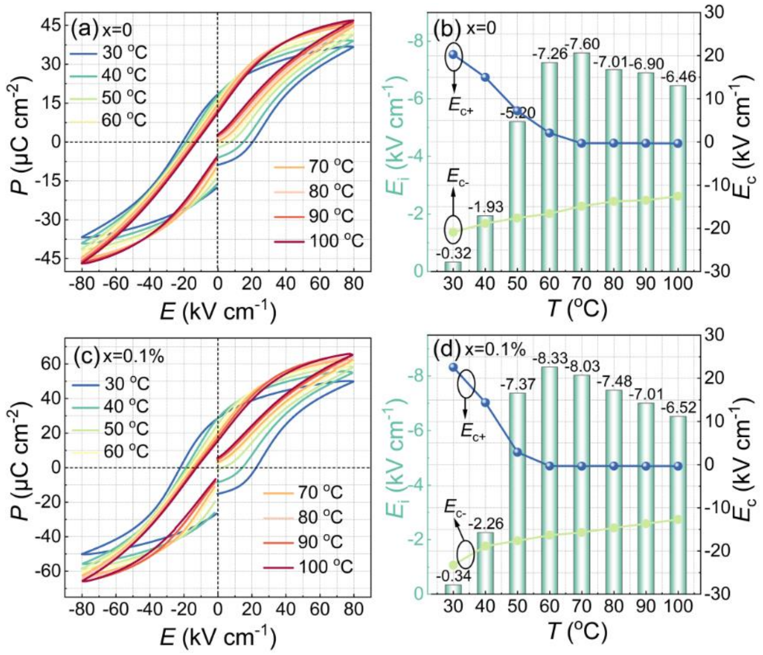

Supplementary Fig. 9 Polarization hysteresis loops measured by Ferroelectric Analyzer System. (a) and (c) The temperature dependence of *P*-*E* loops for pure BNT-BZT and BNT-BZT-*x*GaN with *x*=0.1 wt%. (b) and (d) The temperature dependence of E_{c+}, E_{c-} and E_i for pure BNT-BZT and BNT-BZT-*x*GaN with *x*=0.1 wt%. It can be seen from Fig. S10(a) and (c) that the *P*-*E* loops become pinched and the polarization increases with the increase in temperature. Fig. S10(b) and (d) illustrate positive coercive electric field (E_{c+}), negative coercive electric field (E_{c-}) and internal bias field (E_i) of pure BNT-BZT and BNT-BZT-*x*GaN with *x*=0.1 wt% at the temperature range of 30~100 °C. Here the E_i is calculated according to the equation ($E_i = (E_{c+} + E_{c-})/2$). It can be seen from Fig. S10(b) and (d) that the E_i increases after introducing GaN into BNT-BZT ceramics, which means more defect dipoles act as pinning points in BNT-BZT-*x*GaN with *x*=0.1 wt% to interact with the displacement electric dipoles. Therefore, BNT-BZT-*x*GaN with *x*=0.1 wt% have larger capacity to absorb a greater quantity of opposite electrical charge on both sides of samples in response to temperature change and provide the potential for giant pyroelectric effect.

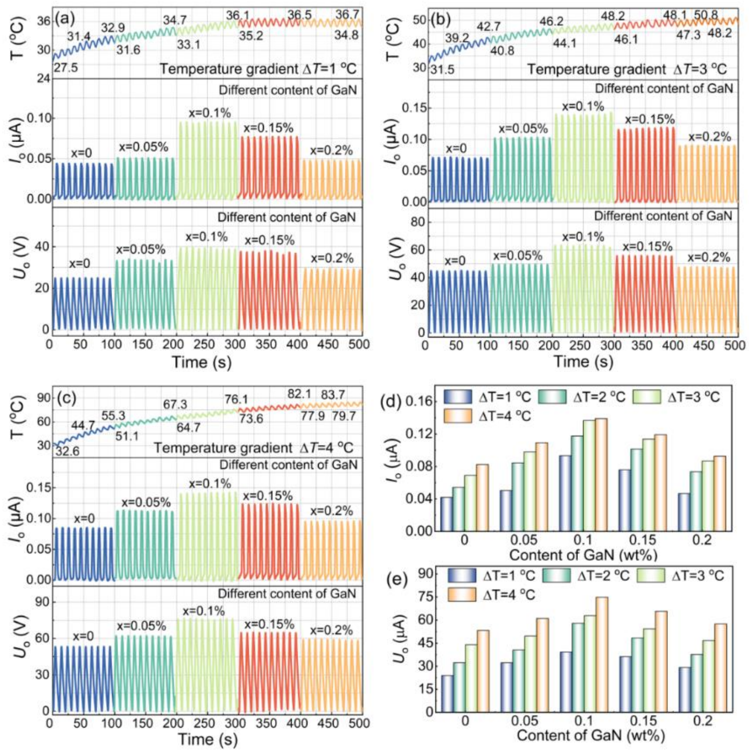

Supplementary Fig. 10 Pyroelectric energy harvesting experimental results measured with the Thermal Energy Harvesting Testing System. (a)-(c) The short-circuit current and open-circuit voltage for BNT-BZT-*x*GaN ceramics with various contents of GaN and ΔT of 1 ºC, 3 ºC, and 4 ºC, respectively. (d)-(e) The comparison of the peak short-circuit current and peak open-circuit voltage values for BNT-BZT-*x*GaN ceramics with ΔT of 1 ºC, 2 ºC, 3 ºC, and 4 ºC, respectively.

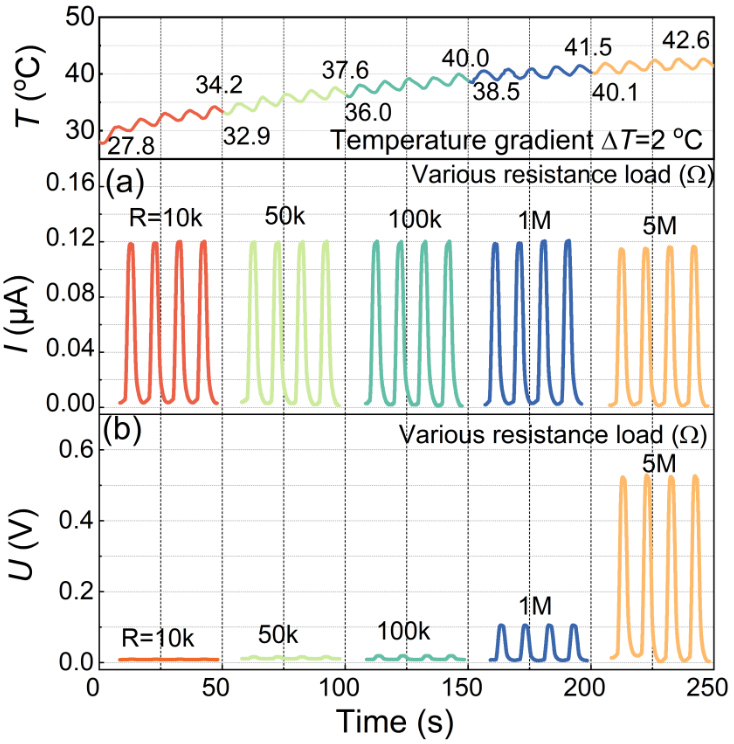

Supplementary Fig. 11 Pyroelectric energy harvesting experimental results measured by the Thermal Energy Harvesting Testing System. (a) and (b) The pyroelectric current and voltage obtained at different load resistances from 10 kΩ to 5 MΩ.

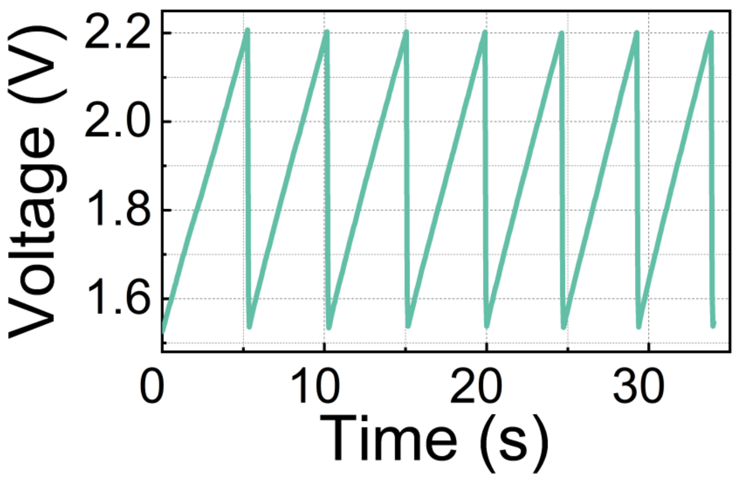

Supplementary Fig. 12 The charging-discharging voltage variation curves of the capacitor driven by BNT-BZT-*x*GaN ceramics with *x*=0.1 wt%.

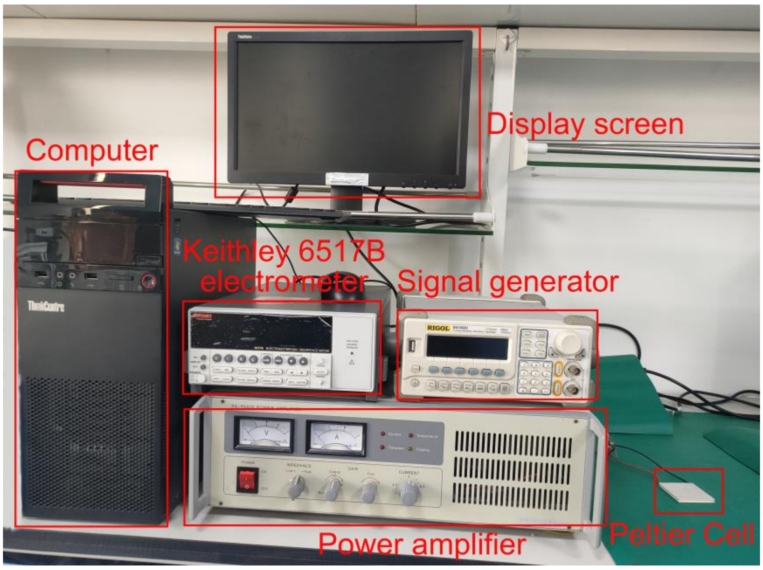

Supplementary Fig. 13 The Thermal Energy Harvesting Testing System: comprising a signal generator, power amplifier, Peltier cell, an electrometer, computer and display screen.
