# Supplementary material for: Thermoelectric coupling effect in BNT-BZT-xGaN pyroelectric ceramics for low-grade temperature-driven energy harvesting
Source: Nat Commun. 2023 Nov 30;14:7907. doi: 10.1038/s41467-023-43692-3 (PMC10689474; doi:10.1038/s41467-023-43692-3)
Supplement: Supplementary file 3 — Description of Additional Supplementary Files [file 41467_2023_43692_MOESM3_ESM.pdf]

Description of Additional Supplementary Files

Supplementary Movie 1: The frequency of TiO_6 vibration.

Supplementary Movie 2: The thermal energy harvesting device lights up the LED bulb.
